# Revascularization character of autologous fascia lata graft following shoulder superior capsule reconstruction by enhanced magnetic resonance imaging

**DOI:** 10.1186/s13018-022-03375-z

**Published:** 2022-11-12

**Authors:** Ya-tao Liao, Huai-sheng Li, Yan Li, Kang-lai Tang, Jing Li, Bing-hua Zhou

**Affiliations:** 1 Department of Sports Medicine, Southwest Hospital, Army Medical University, No. 30 Gaotanyan Main Street, Chongqing, 400038 China; 2 Department of Radiology, Southwest Hospital, Army Medical University, No. 30 Gaotanyan Main Street, Chongqing, 400038 China

**Keywords:** Revascularization, Graft, Superior capsule, Reconstruction, Magnetic resonance imaging

## Abstract

**Background:**

Fascia lata has been used for arthroscopic superior capsule reconstruction (ASCR) and verified to achieve a good clinical outcome. However, it is still not known about revascularization character of the fascia lata after ASCR. This study was performed to evaluate the revascularization of autologous fascia lata grafts after ASCR by enhanced magnetic resonance imaging (MRI).

**Methods:**

A prospective study of 19 patients with irreparable rotator cuff tears underwent ASCR with autologous fascia lata grafts from September 2019 to April 2021. Radiography examinations and clinical evaluations were performed preoperatively and postoperatively at 6 weeks and 3, 6, and 12 months. The signal-to-noise quotient (SNQ) value and enhancement index (EI) of autologous fascia lata grafts in the great tubercle insertion (GTI), midpoint of the graft (MG), and glenoid insertion (GI) were compared for radiography examination. Clinical evaluation included the American Shoulder and Elbow Surgeons (ASES) score, University of California Los Angeles (UCLA) shoulder score, and Visual Analog Scale (VAS) score.

**Results:**

The SNQ values in T1WI enhancement at GI and GTI were significantly higher than those at the plain MRI scan at all postoperative observation timepoints; however, the SNQ values in T1WI enhancement at MG did not show a significant difference until 3 months postoperation. EI values at GTI and GI were significantly higher than those at MG at 6 weeks and 3 months postoperation, while there was no significant difference in the EI value between GTI and GI. At 6 months postoperation, the EI value at GI was significantly higher than those at MG. At 12 months postoperation, the EI value at GI was significantly higher than those at MG and GTI; however, there was no significant difference between GTI and MG. The EI values at GTI and MG peaked at 3 months and 6 months postoperation, respectively, and then plateaued at 12 months postoperation. However, there was no significant difference in the EI value among the different postoperative timepoints at GI. The EI value did not correlate with the VAS and ASES, UCLA scores at any time point or any postoperative observation location.

**Conclusion:**

Revascularization of the fascia lata was dependent on the location of the fascia lata and plateaus at 12 months postoperation. The EI value did not correlate with the VAS and ASES, UCLA scores during12 months postoperation.

## Introduction

Arthroscopic superior capsule reconstruction (ASCR) using autologous fascia lata or dermal allografts has been demonstrated to be a beneficial treatment method for irreparable massive rotator cuff tears [1–3], and ASCR has achieved good short- and medium-term clinical effects [4–8]. ASCR improves shoulder function by restoring the superior stability of the shoulder biomechanically [9]. However, restoring superior stability relies on good fascial bone healing in both the glenoid and rotator cuff footprints, as well as the continuity of the graft.

The fascia lata is rich in blood vessel tissue [10]. Previous research has shown that angiogenesis was important to tendon-to-bone healing [11]. Moreover, angiogenesis was parabolic during the fascial bone healing process in a rat massive rotator cuff model [12]. However, the exact revascularization process of autologous fascia lata grafts following shoulder ASCR is not clearly understood, and there is no clinical report on its evaluation.

Magnetic resonance imaging (MRI) plain scans are the gold standard for the diagnosis of rotator cuff tears [13]; however, plain MRI scans cannot identify blood flow in the graft. Enhanced MRI can display blood flow directly when paramagnetic contrast agents such as Gd-DTPA enter the observed target tissue through blood flow after injection, and the signal values of enhanced MRI changed with the intensity and position of autograft revascularization during the healing process in tendon-to-bone areas [14–17]. However, to date, there has been no radiological evaluation of revascularization of autologous fascia lata grafts after ASCR.

To clarify the characteristics of graft revascularization after ASCR, in this study, we first compared the signal intensity in both plain MRI scans and enhanced MRI to investigate whether enhanced MRI could reveal blood flow in the autogenous fascia lata. Then, we prospectively analyzed the blood circulation in the glenoid, humeral head insertion, and the midpoint of the graft at different time points postoperation.

## Materials and methods

This study was approved by the Ethics Committee of Southwest Hospital (No: KY20202128). From September 2019 to April 2021, the senior author (ZBH) performed all ASCR evaluations in 30 patients, and 19 patients were enrolled in this study (power = 0.8; sig. lever = 0.05; *d* = 0.8). The inclusion criteria were as follows: (1) irreparable massive rotator cuff tears that required ASCR using autologous fascia lata; (2) a follow-up time longer than 12 months; and (3) plain T1WI scans and enhanced MRI performed at all follow-up time points. We defined the exclusion criteria as follows: (1) severe internal diseases, such as heart failure et al.; and (2) graft rupture shown on postoperative MRI.

### MRI scan

All patients were examined by a 1.5 T magnetic resonance scanner (Siemens Magneton Essensa) through a special coil for the shoulder joint. The patients were placed in the supine neutral position, with the opposite injured shoulder joint raised 30°. Then, the palm of the injured arm was placed facing upward, the shoulder was kept in the center of the examination bed as much as possible, and sandbags were laterally placed to prevent movement. An infusion tube was placed in the contralateral anterior elbow vein before examination and rendered the patient completely motionless during examination. Finally, the center was aligned with the coil center and humeral head. The scanning sequence included a plain scan and enhanced scan of the injured shoulder. Plain MRI scan sequences were conducted considering the following parameters: (1) PD-TSE-Dixon coronal position, (2) T1WI-TSE coronal position (scan baseline: parallel to the long axis of the scapula in the transverse position and parallel to the long axis of the humerus in the sagittal position), and (3) T2-TSE-Dixon transverse position. Enhanced MRI sequences were conducted considering the following parameters: (1) T1WI-TSE coronal position (scanning parameters were identical to those of T1WI-TSE plain scanning), (2) T1WI-TSE-FS transverse position, and (3) T1WI-TSE-FS sagittal position (scanning baseline: transverse position ran perpendicular to the long axis of the supraspinatus tendon, while coronal position ran parallel to the long axis of the humerus). Later, an enhanced MRI scan was performed, gadolinium (GdDTPA) contrast agent was injected intravenously at 0.1 mmol/kg, and a T1WI-TSE coronal scan was performed 5 min after injection to achieve the best effect. During the whole data acquisition period, the parameters of the MRI scanning sequences (Table 1) were maintained.

**Table 1:** Parameters of magnetic resonance imaging sequences

Parameter sequences	MRI plain scanning			Enhanced MRI		
	PD-TSE-Dixon coronal position	T1W1-TSE coronal position	T2-TSE-Dixon transverse position	T1W1-TSE coronal position	T1W1-TSE-FS transverse position	T1W1-TSE-FS sagittal position
TR (ms)	3720	600	3000	600	616	724
TE (ms)	38	12	38	12	11	14
Layer thickness (mm)	3	3	3	3	3	3
Layer spacing (mm)	10	10	30	10	30	10
FOV (mm^2^)	180 × 180	180 × 180	180 × 180	180 × 180	180 × 180	192 × 192
Matrix (mm^2^)	256 × 204	256 × 204	256 × 204	256 × 204	256 × 204	256 × 180
Collection numbers	2	2	1	2	1	1
Collection time (min:s)	3:21	2:53	1:53	3:21	1:30	2:15

### MRI measurement

**Fig. 1:**
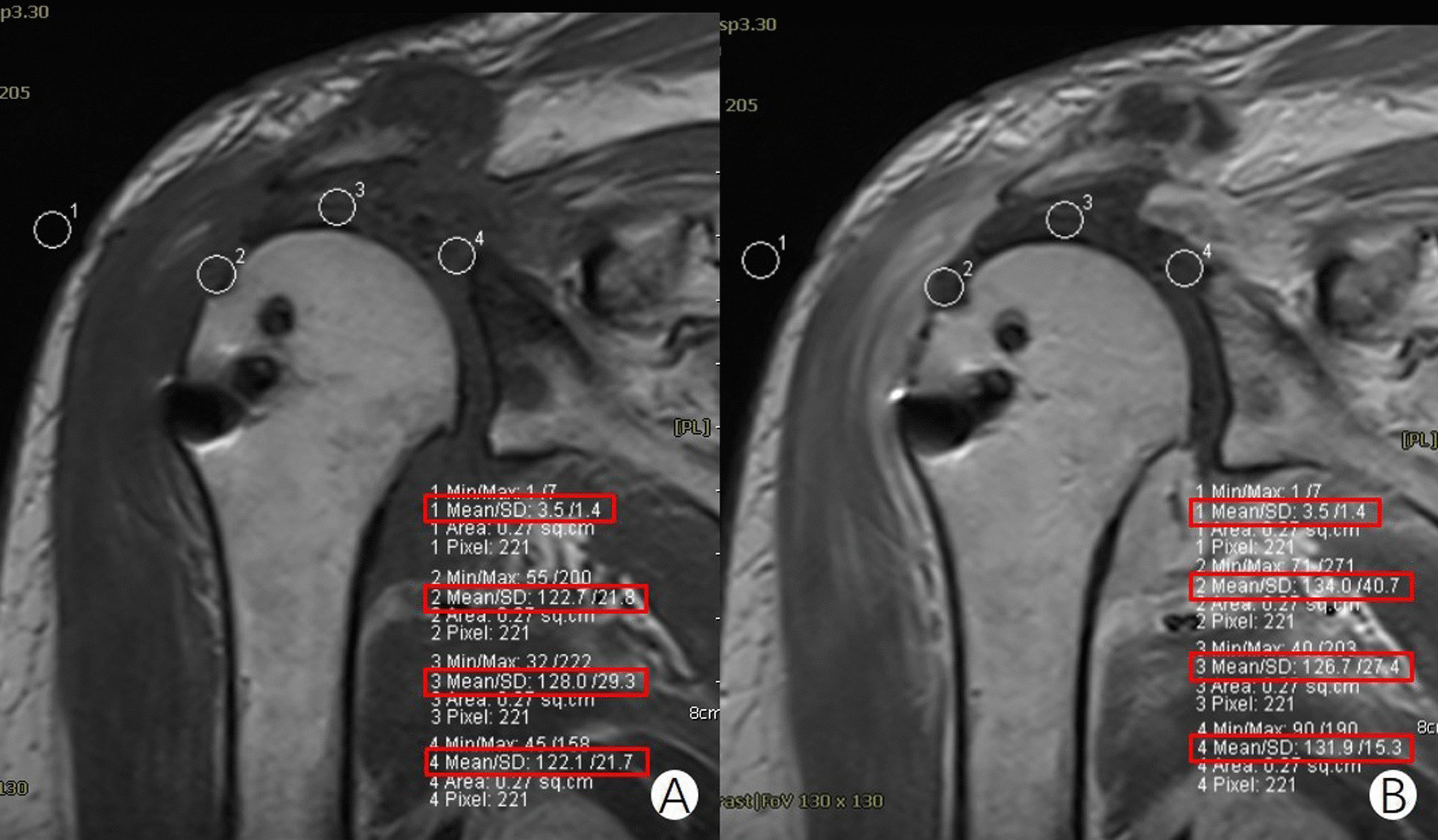
SNQ values were measured in T1WI plain magnetic resonance imaging (MRI) and enhanced MRI scans. **A**: T1WI-TSE-coronal plain scan; **B**: Enhanced T1WI-TSE-coronal position. Red rectangle 1 in both **A** and **B** shows that the signal values in the blank areas of the plain scan and enhancement phases were the same. Red rectangles 2, 3, and 4 show the measurement of signal intensity at three different positions (GTI, MG, and GI)

A Siemens Syngo postprocessing system was used for image analysis. The SNQ values of 5 consecutive slices with good graft integrity were measured on both plain scan and enhanced MRI-T1WI-TSE coronal images. First, the signal value was calibrated by measuring the signal value of the same blank position of the plain T1WI scan and enhancement. Then, the magnetic resonance signal intensities of three parts of the graft (GTI, MG, and GI) were measured (Fig. 1). The FOV selected sites and areas of both plain and enhanced T1WI images were the same. The average value of the 5-slice signal in each FOV region was calculated, and the signal-to-noise ratio (signal-to-noise ratio = measured site signal value/background (blank position) signal value) at 3 points was calculated [15]. Finally, the enhancement index (EI: enhanced SNQ value/plain scan SNQ value) of each point of the graft was calculated. Data measurement and analysis at all selected levels were carried out in exactly the same way. By comparing the changes in signal intensity and EI at the same site between plain scans and enhancement at different time points, indirect information about revascularization was provided to reflect the blood supply of the graft. VAS scores were used to quantitatively score the pain at different postoperative times. ASES and UCLA scores were used to evaluate the postoperative function of the patient at 6 months and 12 months.

### Surgical technique

ASCR was performed using the technique described by Mihata [6, 18]. We performed all procedures using general anesthesia with the patient in the lateral decubitus position. Normal pump pressure was set between 30 and 50 mmHg. We established a posterior portal for initial assessment of the glenohumeral joint and then established an anterior portal through the rotator interval for the treatment of intra-articular lesions, such as labral tears, and repaired the torn subscapularis tendon. We also established a lateral portal and removed any pathologic bursal tissue, performed subacromial decompression to create a flat acromial undersurface and debrided the superior glenoid and rotator cuff footprint of the greater tuberosity to expose cortical bone.

### Measured capsular defect size and harvested fascia lata grafts

The size of the superior was evaluated with a measuring probe in both the anteroposterior (from the anterior edge to the posterior edge of the torn tendon) and mediolateral (from the superior edge of the glenoid to the lateral edge of the greater tuberosity) directions at 30° of shoulder abduction. We made a vertical skin incision over the lateral thigh around the greater trochanter of the femur and harvested the fascia lata. The optimal graft length in the anteroposterior direction was exactly the same as the length of the defect. The graft length in the mediolateral direction was 20 mm longer than the distance from the superior edge of the glenoid to the lateral edge of the greater tuberosity to give a 5-mm footprint on the superior glenoid and a 15 mm footprint on the great tubercle.

### Graft attachment

The graft was fixed to the neck of the glenoid using two anchors (diameter, 5.5 mm, Hearlix, Depuy Mitek, USA) at the 10–11 o’clock and 12–1 o’clock positions in the right shoulder (or the 1–2 o’clock and 11–12 o’clock positions in the left shoulder). The graft was then inserted through the anterolateral portal into the subacromial space directly. When the medial edge of the graft had reached the superior glenoid, all NO. 2 Orthorcords were tied. We attached the lateral side of the fascia lata to the rotator cuff footprint on the greater tuberosity by using the compression double-row technique (2 5.5 mm Healix advance and 2 Versalok). Residual infraspinatus tissue was managed with posterior convergence in all patients after fixation of the graft.

### Postoperative protocol

We recommend the use of an abduction airbag for 6 weeks after reconstruction. After the immobilization period, passive and active-assisted exercises were initiated to promote “scaption” (scapular plane elevation). Three months after surgery, patients began to perform exercises to strengthen the rotator cuff and the scapula stabilizers. Physical therapists assisted all patients.

### Statistical analysis

Statistical analyses were performed with SPSS 22. The average signal values of five layers in each part at different time points were measured, and SNQ and EI values were calculated. Normality of the data sets was assessed using the Kolmogorov–Smirnov and Shapiro–Wilk tests, and appropriate paired Student’s *t* tests or Wilcoxon signed rank tests were then conducted depending on the results of normality testing. The relationship between the VAS, ASES, or UCLA score and EI was analyzed by bivariate correlation analysis.

## Results

### Enhanced MRI could reveal the blood flow in autologous fascia lata grafts after ASCR

To verify that enhanced MRI scans could reveal the revascularization process of the autologous fascia lata grafts, we compared all 19 patients’ MRI plains and enhanced MRI scans of 3 locations at 6 weeks, 3 months, 6 months, and 12 months postoperation. The SNQ values of the autologous fascia lata grafts at GTI, MG, and GI from enhanced MRI examination were significantly higher than those from plain T1WI, except for the comparison at the midpoint of the fascia lata grafts at 6 weeks postoperation (Table 2).

**Table 2:** SNQ values in different positions between the plain scan and the enhanced phase of the T1WI at different time points postoperative

	Greater tubercle			Midpoint of graft			Glenoid insertion		
	T1WI Plain scan	T1WI enhancement	*P* value	T1WI Plain scan	T1WI enhancement	*P* value	T1WI Plain scan	T1WI enhancement	*P* value
6 weeks	27.60 ± 7.59	47.18 ± 22.63	**0.000**	29.20 ± 7.16	39.25 ± 17.95	0.091	29.00 ± 7.32	52.96 ± 23.33	**0.000**
3 months	25.03 ± 5.00	51.07 ± 15.82	**0.000**	26.73 ± 8.20	38.93 ± 13.84	**0.000**	29.15 ± 9.55	55.02 ± 17.97	**0.000**
6 months	28.21 ± 6.67	48.95 ± 10.70	**0.000**	27.11 ± 7.63	45.77 ± 16.78	**0.000**	28.60 ± 6.64	54.57 ± 15.28	**0.000**
12 months	26.69 ± 12.58	40.84 ± 14.40	**0.000**	26.69 ± 12.62	42.75 ± 21.97	**0.000**	26.11 ± 15.60	47.28 ± 23.01	**0.000**

P < 0.05 was statistically significant

### Revascularization of autologous fascia lata grafts was dependent on the different locations

We investigated the revascularization of different locations at the autologous fascia lata grafts at the same timepoint. At 6 weeks and 3 months postoperation, the EI values at GTI and GI were significantly higher than those at MG, while there was no significant difference in the EI value between GTI and GI. At 6 months postoperation, the EI value of the GI was significantly higher than those at MG of the graft. At 12 months postoperation, the EI value on the GI was significantly elevated compared with the MG and GTI (Table 3).

**Table 3:** The comparison of enhancement index in different positions after superior capsule reconstruction

	GT	MG	GI
6 weeks	1.64 ± 0.45^#^	1.33 ± 0.47	1.76 ± 0.49^#^
3 months	2.06 ± 0.49^#^	1.53 ± 0.53	1.92 ± 0.38^#^
6 months	1.77 ± 0.31	1.67 ± 0.40	1.91 ± 0.35^#^
12 months	1.61 ± 0.33	1.63 ± 0.41	1.88 ± 0.29^*#^

GT: Greater tubercle; MG: midpoint of graft; GI: glenoid insertion

^*^ Compared with GT, *P* < 0.05; ^#^ Compared with MG, *P* < 0.05

### Revascularization of autologous fascia lata grafts at GTI and MG was time-dependent; however, revascularization at GI was under high level consistently during 12 months postoperation

We compared the EI of autologous fascia lata grafts at different time points after ASCR to analyze the change in graft revascularization at the same location over time. At the great tubercle, the EI value first increased and peaked at 3 months, then decreased and finally plateaued at 12 months postoperation. The EI showed a significant increase at 3 months postoperation compared with that at 6 weeks postoperation. Compared with 3 months postoperation, there was a significant decrease at 6 months and 12 months postoperation, while no significant difference between 12 and 6 months postoperation was detected.

At the midpoint of the graft, the EI value peaked at 6 months postoperation and plateaued at 12 months postoperation. The EI value was significantly increased at 6 and 12 months compared with 6 weeks postoperation. Compared with 3 months postoperation, the EI at 6 months and 12 months postoperation was no significant difference; however, there was no significant difference between those at 6 months and 12 months postoperation.

**Fig. 2:**
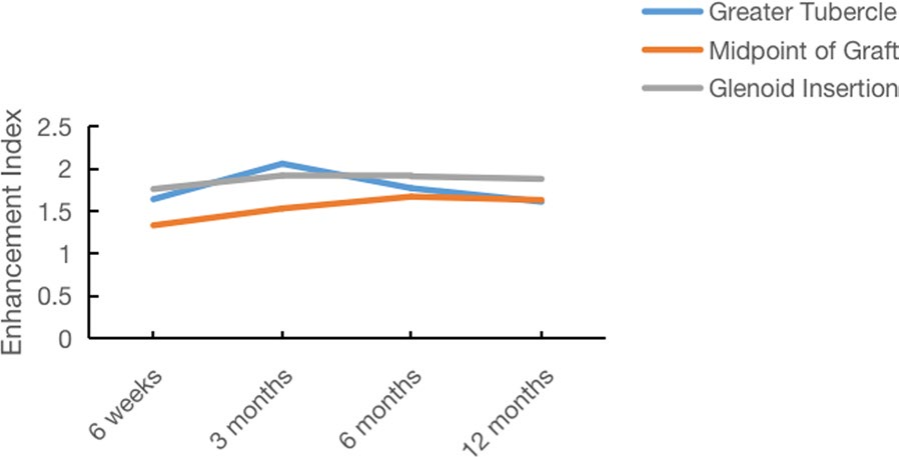
Comparison of the enhancement index of the graft at different time points at the great tubercle, the midpoint of the graft and the glenoid insertion

At glenoid insertion, there was no significant difference in the EI value among the different timepoints. No significant difference was shown in the EI value among 6 weeks and 3, 6 and 12 months postoperation (Table 4 and Fig. 2).

**Table 4:** The comparison of enhancement index value at different time points after superior capsule reconstruction

	6 weeks	3 months	6 months	12 months
GT	1.64 ± 0.45	2.06 ± 0.49^α^	1.77 ± 0.31^β^	1.61 ± 0.33^β^
MG	1.33 ± 0.47	1.53 ± 0.53	1.67 ± 0.40^α^	1.63 ± 0.41^α^
GI	1.76 ± 0.49	1.92 ± 0.38	1.91 ± 0.35	1.88 ± 0.29

GT: Greater tubercle; MG: midpoint of graft; GI: glenoid insertion

^α^Compared with 6 weeks postoperative, *P* < 0.05; ^β^Compared with 3 months postoperative, *P* < 0.05; ^γ^Compared with 6 months postoperative, *P* < 0.05

### Correlation between shoulder function and revascularization of autologous fascia lata grafts

The VAS was used to evaluate the degree of postoperative pain, the ASES and UCLA were used to evaluate postoperative functional recovery, and the correlation between the VAS, ASES, or UCLA score and the EI at different time points and different parts was analyzed. The results showed no significant correlation between the VAS scores and EI value at any time point and any observation location (Table 5). Also, the results showed no significant correlation between the ASES and UCLA scores and EI value at any time point and any observation location (Table 6).

**Table 5:** The correlation between VAS and EI value at different time points and different parts

	VAS score	EI value					
		GT		MG		G	
		Spearman	*P*	Spearman	*P*	Spearman	*P*
VAS							
6 weeks	3.63 ± 1.06	-0.094	0.825	-0.445	0.270	-0.021	0.960
3 months	2.67 ± 0.51	-0.454	0.365	-0.808	0.052	-0.203	0.700
6 months	1.43 ± 0.53	0.275	0.550	-0.374	0.409	0.126	0.787
12 months	1.12 ± 0.35	0.065	0.879	-0.560	0.149	-0.014	0.974

GT: Greater tubercle; MG: midpoint of graft; GI: glenoid insertion

*P* < 0.05 was statistically significant

**Table 6:** The correlation between ASES, UCLA, and enhancement index at 6 months and 12 months after operation

	EI value					
	GT		MG		G	
	Spearman	*P*	Spearman	*P*	Spearman	*P*
ASES						
6 months	− 0.396	0.379	0.682	0.085	0.074	0.875
12 months	− 0.112	0.792	0.562	0.147	− 0.109	0.798
UCLA						
6 months	− 0.365	0.420	0.301	0.512	0.146	0.755
12 months	− 0.112	0.773	0.444	0.271	− 0.238	0.571

GT: Greater tubercle; MG: midpoint of graft; GI: glenoid insertion

*P* < 0.05 was statistically significant

## Discussion

ASCR has been suggested to be a clinical choice for irreparable massive rotator cuff tears [5, 19]. The aim of ASCR is to restore the superior stability of the shoulder biomechanically, and the most important aspect is to achieve good fascial bone healing in both the glenoid insertion and rotator cuff footprint. Autograft revascularization is a key factor for fascial bone healing and good clinical outcome. Therefore, we evaluated the revascularization of fascia lata grafts after ASCR through enhanced MRI.

Enhanced MRI can evaluate revascularization in fascia lata grafts after ASCR. As a noninvasive imaging method, MRI has been widely used in the diagnosis of different types of shoulder diseases. It can not only be used in the diagnosis of rotator cuff tears but also reflect the blood supply of rotator cuffs by comparing the changes in magnetic resonance SNQ values [20]. Sasanuma H. found that shoulder pain and limited movement were related to abnormal hemodynamics by dynamic magnetic resonance imaging [21, 22]. Kim found inferior tendon perfusion immediately after repair [14]; however, there was no long-term observation. In this study, we found that the T1WI enhancement signal values of the fascia lata grafts were significantly higher than those of the plain scan at different postoperative timepoints, except at the midpoint of the fascia lata 6 weeks postoperation. We reasonably assert that revascularization of the fascia lata at the GI and GTI appeared at 6 weeks postoperation, but not at the MG. Enhanced MRI can be used to evaluate the revascularization of fascia lata grafts after ASCR.

The revascularization of the fascia lata changed over time. The EI of the fascia lata in the great tubercle increased significantly at 6 weeks postoperation and peaked at 3 months postoperation, and there was no significant difference between 6 and 12 months postoperation. This finding supported that angiogenesis was parabolic and progressed downward during the fascial bone healing process in a rat massive rotator cuff model [12]. Angiogenesis might be helpful for the healing of the fascia-to-bone interface in the early phase. When healing was complete, inflammation and angiogenesis receded. The EI of the fascia lata at the MG peaked at 6 months postoperation and plateaued at 12 months postoperation, which means that revascularization was late compared at the GTI and GI. However, at the GI, there was no significant difference in the EI value among the different timepoints. Therefore, it is reasonable to speculate that the revascularization of the fascia lata at the GI was stable in the early stage of ASCR. In summary, revascularization of the fascia lata was time-dependent, and revascularization of the fascia lata first appeared at the GI and GTI and then extended to the MG.

The biomechanical basis of ASCR is to restore the superior stability of the shoulder joint through better fascia-to-bone healing. Benke et al. believed that in the surgical repair of rotator cuff tears, simple suturing of the broken end should not be adopted. Instead, the ischemic tissue of the broken end should be excised, and then sutured or appropriate tendon substitute materials should be used for repair, which is conducive to healing and better long-term efficacy by improving the local blood supply [23]. In this study, we found that the revascularization of the fascia lata was location-dependent. The revascularization of the autogenous fascia lata grafts was first established at GTI and GI and then extended to the MG. Revascularization of the fascia lata grafts at the GI reached a stable state at 6 weeks postoperation; however, at the GTI and MG, it plateaued 12 months postoperation. Ntoulia A.F. found that graft revascularization first appeared in the intra-articular part and then extended to other parts of the graft after anterior cruciate ligament reconstruction and speculated that the process of revascularization was also closely related to the surrounding microenvironment [15]. Harukazu further confirmed that vascular epithelial growth factor (VEGF) played an important role in the process of revascularization [24]. Collectively, the results of this study suggested that revascularization of the fascia lata after ASCR might be formed at the GI and GTI first and then extend to MG. The revascularization of the fascia lata grafts was location-dependent and obviously affected by multiple factors, such as the local microenvironment and biological factors, but further research is needed.

Different augmentation implants, grafts, or scaffolds will be used for compromised rotator cuff tissue quality. Bio-inductive collagen implant was introduced for augmenting rotator cuff repair [24] and chronic tendinopathy [25]. Muench reported that subacromial bursa-derived cells and concentrated bone marrow aspirate demonstrated high cellular adhesion and proliferation potential on demineralized bone matrix scaffolds [26], which indicated that stem cell could be used for improving arthroscopic rotator cuff repair. However, there were still not long-term and high-quality comparative studies for both bio-inductive collagen implant and stem cell therapy for rotator cuff tear. In a word, stem cell therapy is very promising and exciting to use stem cells in the near future.

Different graft materials were used for SCR including in teflon felt synthetic graft [18], semitendinosus tendon autograft [27], biceps tendon [28]. Dukan et al. reported that SCR with a porcine dermal matrix xenograft improved shoulder outcome and restored the acromiohumeral distance [29]. In fact, both of fascia lata and double-layer porcine dermal matrix xenograft may restore superior translation and subacromial contact pressure [30]. That is why different grafts could achieve a good clinical result biomechanically. Regardless of success in the long term after SCR, the chief concern is to attain graft bone healing in both the glenoid and rotator cuff footprint [19]. Revascularization of the graft also probably affected the complication rate. The overall complication rate post-SCR ranged from 5.0 to 70.0%, and with allograft 19–70% and autograft 8–29%, respectively [31]. The complication rate of the SCR with porcine dermal graft was higher compared with autologous fascia lata graft. In spite of availability, harvest-site morbidity, cost, and mechanical strength, more attention should pay on the revascularization of the graft; however, the revascularization of porcine dermal graft is limited.

This study has several limitations. First, only 19 patients were included in this study. However, this study was designed to be a prospective study, and power analysis showed that 19 patients were sufficient. Second, we did not show the vessels in the fascia lata graft directly; however, revascularization was evaluated by the SNQ value and EI value at different time intervals and 3 different locations of the graft, and we believe the results are still robust. Further study and new techniques are needed to reveal the angiogenesis process and blood supply in fascia lata grafts directly.

In conclusion, MRI enhancement was successfully used to evaluate revascularization of the fascia lata after ASCR. Revascularization of the graft first formed at the great tuberosity and glenoid and then extended to the midpoint of the graft. Revascularization of the fascia lata grafts at the glenoid insertion reached a stable state at 6 weeks postoperation; however, at the great tubercle and midpoint of the graft, it plateaued 12 months postoperation. The EI value did not correlate with the VAS and ASES, UCLA scores during12 months postoperation.

## Data Availability

The data sets used and/or analyzed during the current study are available from the corresponding author on reasonable request.

## Abbreviations

SCR: Superior capsule reconstruction
ASCR: Arthroscopic superior capsule reconstruction
GTI: Great tubercle insertion
MG: Midpoint of the graft
GI: Glenoid insertion
MRI: Magnetic resonance imaging
SNQ: Signal-to-noise quotient
EI: Enhancement index
ASES: American Shoulder and Elbow Surgeons
UCLA: University of California Los Angeles
VAS: Visual Analog Scale
VEGF: Vascular epithelial growth factor
